# Feasibility of Switching to an Integrase Inhibitor-Based Single-Tablet Regimen in Adults Living With HIV and Sustained Virological Suppression on Outdated Antiretroviral Therapies

**DOI:** 10.7759/cureus.98987

**Published:** 2025-12-11

**Authors:** Giorgio Tiecco, Maria Alberti, Martina Salvi, Irene Scarvaglieri, Federico Cesanelli, Emanuele Focà, Francesco Castelli, Eugenia Quiros-Roldan

**Affiliations:** 1 Department of Clinical and Experimental Sciences, University of Brescia, Brescia, ITA

**Keywords:** antiretroviral therapy, outdated antiretroviral regimens, outdated art, outpatient care, real-life, real-world

## Abstract

Background

Despite advances in antiretroviral therapy (ART), a subset of people living with HIV (PLWH) remain on outdated regimens (OUT-ART), which are associated with higher toxicity, reduced tolerability, and increased pill burden. We conducted a cross-sectional, observational study at a tertiary HIV clinic in Northern Italy to assess the prevalence, clinical characteristics, and switch feasibility of PLWH on OUT-ART.

Methods

The study enrolled all PLWH currently receiving OUT-ART who had attended at least one clinical visit between January 2023 and January 2024 at the HIV outpatient clinics of the Infectious Diseases Unit, Azienda Socio Sanitaria Territoriale (ASST) Spedali Civili di Brescia in Brescia, Italy. Eligible participants were aged ≥18 years, had sustained virological suppression (HIV-RNA < 50 copies/mL), and were receiving ART regimens not listed as first-line or alternative options in the 2023 EACS guidelines. Participants were categorized into two groups: those for whom a switch to an integrase inhibitor-based single-tablet regimen (INI STR) was feasible (Group A) and those for whom it was not (Group B). Demographic, clinical, resistance, and adverse drug reaction (ADR) data were analyzed using descriptive statistics. To evaluate factors associated with the feasibility of switching to an INI-based STR and its relationship with ADR, we performed multivariate logistic regression with multiple imputation and Firth’s correction on normalized data.

Results

Among 3,848 PLWH in care, 54 (1.4%) were on OUT-ART, and 40 (1.0%) met inclusion criteria: Group A comprised 22 individuals; Group B, 18. Our sample had a mean age of 55.4 (± 9.2) years and a mean duration of HIV infection of 24.1 (± 8.8) years, with a generally preserved immunological profile. Genotypic resistance analysis revealed a high prevalence of thymidine analogue mutations in both groups, with non-significantly higher rates of K65R, M184V. INI-resistance mutations were rare. Mild-to-moderate biochemical ADRs were frequent in both groups (63.6% vs. 72.2%, p=0.564), with Group B showing significantly higher rates of hyperbilirubinemia (27.8% vs. 4.5%, p=0.041) and metabolic alterations (27.8% vs. 0%, p=0.008). Multivariate analysis showed that patient refusal was the only variable significantly associated with continued OUT-ART use despite eligibility for switching (OR=581.8; 95% CI: 2.5-134035.4; p=0.0279).

Conclusions

Patient preference, rather than clinical or virological factors, was the main barrier to regimen optimization. These findings emphasize the need for shared decision-making strategies to improve care in aging, treatment-experienced HIV populations.

## Introduction

The evolution of antiretroviral therapy (ART) has profoundly transformed the management of people living with HIV (PLWH), enabling sustained virologic suppression, improved immune restoration, and significant reductions in HIV-associated morbidity and mortality [1]. Consequently, these advancements have greatly enhanced the quality of life for PLWH [2]. Current international guidelines, including those from the European AIDS Clinical Society (EACS) published in 2025, recommend ART regimens that prioritize high tolerability, a robust genetic barrier to resistance, and simplicity in administration [3]. Integrase strand transfer inhibitor (INI)-based regimens have become the cornerstone of first- and second-line treatments due to their optimal safety profiles and efficacy, reinforcing the goal of maintaining long-term virologic control while minimizing adverse effects [3].

Despite the availability of highly effective and tolerable ART options, some PLWH continue to receive obsolete ART regimens (OUT-ART). These regimens include older drugs such as nevirapine, atazanavir, etravirine, maraviroc, and lopinavir, which are associated with higher risks of adverse effects, including hepatotoxicity, gastrointestinal disturbances, and cardiovascular complications. Furthermore, these agents are not available as part of a single-tablet regimen (STR), thereby potentially compromising treatment adherence [4-8]. Therefore, OUT-ART regimens fail to align with modern therapeutic standards, as they can compromise adherence, elevate the risk of therapeutic failure, and contribute to the accumulation of drug resistance. Moreover, their continued use exacerbates the management of comorbidities prevalent in aging PLWH, such as dyslipidemia, hypertension, and renal impairment mortality [1,9]. Transitioning these patients to contemporary regimens is complicated by factors such as clinical complexity, patient reluctance, and limited therapeutic alternatives in some cases [10].

To address the clinical gap in the management of PLWH receiving OUT-ART, we aim to investigate the prevalence and impact of these regimens within our cohort of PLWH under care at the HIV outpatient clinics of the Infectious Diseases Unit of a tertiary hospital in Northern Italy. Furthermore, we analyze demographic, clinical, and viro-immunological differences between PLWH receiving OUT-ART who are eligible for switching to an INI-based STR and those for whom such a switch is not feasible, with particular emphasis on adverse drug reactions (ADR) and reasons for maintaining OUT-ART.

## Materials and methods

Study design and participants

This observational, cross-sectional, single-center study was conducted at the HIV outpatient clinics of the Infectious Diseases Unit, Azienda Socio Sanitaria Territoriale (ASST) Spedali Civili di Brescia, in Brescia, Italy. The study enrolled all PLWH currently receiving OUT-ART who had attended at least one clinical visit between January 2023 and January 2024. Inclusion criteria encompassed individuals aged 18 years or older with sustained virological suppression (HIV-RNA < 50 copies/mL) currently receiving ART regimens not recommended as first-line or alternative options according to the 2023 EACS guidelines. Additional eligibility requirements included the availability of at least one genotypic resistance test and completion of the most recent follow-up visit within the study period. Informed consent was obtained from all participants. No further exclusion criteria were applied. Participants were stratified into two groups: those on OUT-ART who were eligible for a feasible switch to an INI-based STR (group A) and those for whom such a switch was not feasible (group B).

Definitions

OUT-ART regimens were defined as ART treatments that are no longer recommended or listed as first-line or alternatives in the EACS 2023 guidelines [3]. ADRs were defined according to the most frequently reported ADRs listed in the OUT-ART leaflet. They were classified as 'clinical' when characterized by observable signs or symptoms directly attributable to medication use and as 'biochemical' when identified through laboratory abnormalities associated with the drug. Metabolic alterations were defined as the presence of hypercholesterolemia, hypertriglyceridemia, or dyslipidemia, as identified through laboratory assessments. A virologic blip was defined as an isolated HIV-1 RNA value of 50-999 copies/mL, flanked by values below assay detection limits (<50 copies/mL) [11]. A virological failure was defined as either two consecutive HIV-1 RNA measurements > 200 copies/mL following interval adherence counseling or a single HIV-1 RNA measurement > 1000 copies/mL [12]. “MegaART” was defined as a regimen consisting of more than three antiretroviral agents. All resistance-associated mutations and polymorphisms were reviewed in accordance with the most recent version of the Stanford University HIV Drug Resistance Database [13]. The feasibility of switching to an INI STR was evaluated based on cumulative genotypic resistance testing and documented history of prior intolerance, either reported or confirmed, to INI.

Data collection

Data were collected using a structured database developed in REDCap (Vanderbilt University, Nashville, TN), ensuring pseudonymization for patient confidentiality [14]. Variables included demographic and general patient characteristics (age, sex, HIV diagnosis year, history of virologic failures), immunologic and virologic data (nadir CD4+, CD4+/CD8+ ratio, quantitative HIV-RNA, and historical resistance genotypes), and ART-specific data (current regimen, prior switches, and availability of alternative regimens). Outcomes such as ADRs, blips, and virologic failure were also documented. Data were sourced from electronic medical records and supplemented with historical HIV-1 resistance testing evaluated through the HIV Drug Resistance Database [13].

Ethics

The study protocol was approved by the Territorial Ethics Committee Lombardy 6, Pavia, Italy (protocol number 0018047/25), and conducted in accordance with the ethical standards of the Helsinki Declaration (1975, revised in 2013). Written informed consent has been obtained from each subject.

Statistical analysis

The dataset was stratified into two groups: PLWH receiving OUT-ART who were eligible for switching to an INI-based STR (group A) and those for whom such a switch was not feasible (group B) based on therapeutic history, genotype resistance test, and deposed intolerance to INI. Categorical variables were summarized as frequencies and percentages, while continuous variables were reported as means with standard deviations. Continuous non-parametric variables were compared using the Mann-Whitney U. Categorical variables were analyzed using the chi-squared test or Fisher’s exact test when expected cell counts were small. The primary endpoint, proportion of ADRs among individuals with or without a feasible switch option, was assessed using the chi-squared test. To perform regression analysis, the dataset was initially pre-processed. Missing data were handled using multiple imputation by chained equations (MICE). Predictive mean matching (PMM) was chosen as the imputation method [15]. The imputation model included all variables used in the subsequent analyses. Five imputed datasets (m = 5) were generated with 50 iterations each to ensure convergence of the MICE algorithm. Diagnostics based on trace plots did not show evidence of non-convergence. To account for differences in scale among continuous variables (viral load zenith and nadir CD4 count), min-max normalization was performed, standardizing values to a (0,1) range [16], ensuring consistent scaling for analysis. Univariate and multivariate logistic regression models were fitted separately on each imputed dataset to assess associations with the feasibility of the switch to INI-based STR. Given quasi-complete separation for some variables, Firth’s bias-reduced logistic regression was used to ensure stable estimations [17]. The multivariate model adjusted for potential confounders, including sex, age, multimorbidity, smoking status, alcohol consumption, duration of current ART regimen (in months), viral load zenith, nadir CD4 count, and reason for not switching to INI-based STR. Collinearity among covariates was assessed using the variance inflation factor (VIF, with a <5 threshold). Pooled estimates and standard errors were computed using Rubin’s rules [18]. Results are presented as pooled ORs with 95% CIs. Due to the limited availability of prior data, a formal sample size calculation could not be conducted; thus, the study was conceived as a pilot investigation. All statistical analyses were performed using Jamovi (The jamovi project (2023), version 2.3, retrieved from https://www.jamovi.org) and R statistical software (version 4.3.1, The R Core Team, R Foundation for Statistical Computing, Vienna, Austria) [19,20], with a significance threshold set at α < 0.05.

## Results

Our HIV outpatient clinics routinely and actively follow 3,848 PLWH, most of them on modern ART, mostly bictegravir/emtricitabine/tenofovir alafenamide (1449/3848, 37.7%) and dolutegravir/lamivudine (1182/3848, 30.7%). A total of 52 (52/3848, 1.4%) individuals were screened for eligibility, based on their sustained virological suppression while on OUT-ART. Of these, 12 participants were excluded: three due to lack of follow-up within the prior 12 months and nine due to the absence of genotypic resistance testing. The final study cohort comprised 40 individuals, stratified into two groups: 22 PLWH in group A and 18 in group B.

Our sample had a mean age of 55.4 (± 9.2) years and a mean duration of HIV infection of 24.1 (± 8.8) years, with a generally preserved immunological profile characterized by a mean CD4/CD8 ratio of 0.80 ± 0.37 (CD4 count of 769 ± 327 cells/mcL and CD4% of 29.3 ± 8.9). Demographical and clinical information are extensively reported in Table 1, but no significant statistical difference was found between group A and group B.

**Table 1:** Demographic and clinical characteristics of the study group All percentages were calculated based on available data. INI: integrase inhibitors; STR: single tablet regimen; HBV: hepatitis B virus; HCV: hepatitis C virus; PWID: people who inject drugs

Sample	Group A: Feasible INI STR switch	Group B: Non-feasible INI STR switch	p-value
Total included, n (%)	22 (100)	18 (100)	
Demographic			
Female, n (%)	11 (50)	10 (55.6)	0.726
Age, in years, mean (±SD)	54.9 (6.89)	55.9 (11.4)	0.870
European origin, n (%)	16 (72.7)	13 (72.2)	0.972
Comorbidities			
Smoking, n (%)	8 (50)	5 (31.3)	0.280
Hypertension, n (%)	11 (50)	11 (61.1)	0.482
Diabetes, n (%)	1 (4.5)	4 (22.2)	0.093
Dislipidaemia, n (%)	15 (68.2)	11 (61.1)	0.641
Current HBV infection, n (%)	9 (40.9)	6 (33.3)	0.622
Previous HCV infection, n (%)	9 (40.9)	5 (27.8)	0.386
Past or current alcohol abuse, n (%)	0 (0)	2 (12.5)	0.157
Ex or current PWID, n (%)	4 (21.1)	5 (31.3)	0.492
Previous or current cancer, n (%)	4 (18.2)	3 (16.7)	0.900
Psychiatric disorders, n (%)	5 (22.7)	4 (22.2)	0.970
Multimorbidity, n (%)	11 (52.4)	10 (55.6)	0.843
Polypharmacy, n (%)	11 (52.4)	12 (66.7)	0.366

Similarly, OUT-ART regimens were broadly comparable between groups, with no significant differences observed in the use of anchor drugs, pill burden, dosing frequency, or viro-immunological characteristics (Table 2).

**Table 2:** OUT-ART regimens, and viro-immunological characteristics. All percentages were calculated based on available data. INI: integrase inhibitors; STR: single tablet regimen; OUT-ART: outdated antiretroviral therapy; ART: antiretroviral therapy; VS: virological suppression

Sample	Group A: Feasible INI STR switch	Group B: Non-feasible INI STR switch	P-value
Total included, n (%)	22 (100)	18 (100)	
OUT-ART			
Atazanavir-containing regimen, n (%)	14 (63.6)	8 (44.4)	0.225
Etravirine-containing regimen, n (%)	7 (31.8)	5 (27.8)	0.781
Maraviroc-containing regimen, n (%)	2 (9.1)	4 (22.2)	0.247
Nevirapine-containing regimen, n (%)	1 (4.5)	1 (5.6)	0.884
Mega-ART, n (%)	2 (9.0)	3 (1.7)	0.471
Number of pills/die, mean (±SD)	2.5 (0.7)	2.3 (0.7)	0.482
Number of administrations/die, mean (±SD)	1.45 (0.5)	1.56 (0.5)	0.541
Month on OUT-ART, mean (±SD)	121 (65.9)	107 (67.1)	0.714
Month with VS on OUT-ART, mean (±SD)	103 (72.8)	103 (57.2)	0.644
Blip occurred during OUT-ART, n (%)	6 (27.3)	8 (44.4)	0.257
HIV initial infection			
Subtype B, n (%)	9 (75.0)	5 (62.5)	0.550
Years from HIV infection, mean (±SD)	24.1 (9.03)	24.1 (8.52)	0.978
Log₁₀-HIV-RNA zenit, mean	5.32 (0.37)	5.30 (0.23)	0.687
CD4 nadir, mean (±SD)	168 (191)	175 (109)	0.286
Last visit immunological profile			
CD4 cells count, mean (±SD)	814 (319)	714 (330)	0.355
CD4%, mean (±SD)	29.7 (9.05)	28.9 (8.73)	0.754
CD8 cells count, mean (±SD)	1137 (580)	1092 (624)	0.860
CD8%, mean (±SD)	39.6 (12.7)	42.5 (11.2)	0.341
CD4/CD8 ratio, mean (±SD)	0.832 (0.382)	0.763 (0.358)	0.568

As regards genotypic resistance data, the prevalence of at least one thymidine analogue mutation (TAM) was high in both groups (88.2% vs. 83.3%, p = 0.706). Although not statistically significant, the K65R mutation was more frequently observed in group B (42.9% vs. 13.3%; p = 0.075), as was the M184V mutation (78.6% vs. 60.0%; p = 0.280). Major PI-associated mutations (L90M and I84V) were more frequent in group B, though differences were not statistically significant. INI-resistance mutations were rare, with only one participant in each group harboring a relevant mutation (Q148 or N155H) (5.0% vs. 7.7%, p = 0.751).

As shown in Table 3, no clinical ADRs were detected in either group; however, the prevalence of mild-to-moderate ongoing biochemical ADRs was high in both groups, with no significant difference (63.6% in group A vs. 72.2% in group B, p = 0.564). Hyperbilirubinemia (27.8% vs. 4.5%, p = 0.041) and metabolic alterations (27.8% vs. 0%, p = 0.008) were significantly more frequent in group B. Patient refusal to change was the main reason for non-switching in group A (54.5% vs. 16.7%, p = 0.013), whereas previous INI intolerance was reported exclusively in group B (0% vs. 38.9%, p < 0.01).

**Table 3:** Adverse effects and reasons not to switch All percentages were calculated based on available data INI: integrase inhibitors; STR: single tablet regimen; OUT-ART: outdated antiretroviral therapy; ADRs: adverse drug reactions

Sample	Group A: Feasible INI STR switch	Group B: Non-feasible INI STR switch	P-value
Total included, n (%)	22 (100)	18 (100)	
Adverse effects of OUT-ART			
Clinical ADRs	0 (0.0)	0 (0.0)	
Biochemical ADRs	14 (63.6)	13 (72.2)	0.564
Mild-to-moderate, n (%)	14 (63.6)	13 (72.2)	0.564
Hepatic toxicity, n (%)	7 (31.8)	3 (16.7)	0.271
Hyperbilirubinemia, n (%)	1 (4.5)	5 (27.8)	0.041
Metabolic alterations, n (%)	0 (0.0)	5 (27.8)	0.008
Other ADRs, n (%)	6 (27.3)	0 (0.0)	0.011
Severe, n (%)	0 (0.0)	0 (0.0)	
Reasons not-to-switch			
Patient refusal, n (%)	12 (54.5)	3 (16.7)	0.013
INI intolerance, n (%)	0 (0.0)	7 (38.9)	<0.01
Unknown, n (%)	10 (45.5)	8 (44.4)	0.951

**Figure 1:**
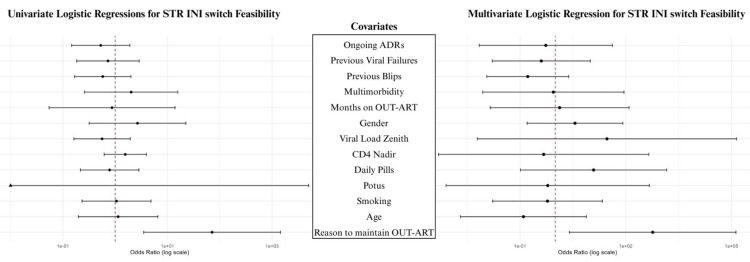
Forest plots for univariate and multivariate logistic regression analysis STR: single tablet regimen; INI: integrase inhibitors; ADRs: adverse drug reactions; OUT-ART: outdated-antiretroviral therapies

In the primary analysis, no associations between the studied variables and maintaining OUT-ART despite the feasibility of a switch to INI-based STR were observed. In particular, in both univariate and multivariate logistic regression models, the association between switch feasibility to an INI-based STR (as dependent variable) and the ongoing mild-to-moderate biochemical ADRs (univariate OR: 0.5292, 95% CI: 0.1468-1.9075, p = 0.3326; multivariate OR: 0.5372, 95% CI: 0.0069-41.7676, p = 0.7868), previous viral failure (univariate OR: 0.7274, 95% CI: 0.1838-2.8786, p = 0.6503; multivariate OR: 0.4005, 95% CI: 0.0163-9.8505, p = 0.5769), and previous blips (univariate OR: 0.5782, 95% CI: 0.1673-1.9985, p = 0.3868; multivariate OR: 0.1649, 95% CI: 0.0113-2.4032, p = 0.1916) were not statistically significant. The only variable showing statistically significant association with the possibility of switching to INI-based STR while on OUT-ART was the patient's refusal to change regimen (univariate OR: 71.1930, 95% CI: 3.5063-1445.5111, p = 0.0075; multivariate OR: 581.8069, 95% CI: 2.5254-134035.3902, p = 0.0279) (Figure 1, Appendix A, Appendix B).

## Discussion

Although our tertiary care center hosts one of the largest cohorts of PLWH in Italy, with broad access to modern ART, including long-acting injectables (LAIs) and therapies reserved for highly treatment-experienced (HTE) individuals, 1.4% of patients remain on OUT-ART. This study offers a real-world characterization of a persistent global challenge, providing comprehensive clinical, virological, and immunological data on this vulnerable subgroup. Since remaining on OUT-ART despite the feasibility of switching to an INI-based STR was not statistically associated with ongoing biochemical ADRs, previous treatment failure, or previous virologic blips, our findings underscore that clinical decision-making in this context is often guided by patient preference and individuals’ circumstances rather than virological or safety constraints alone.

A high prevalence of mild-to-moderate biochemical ADRs was observed in both groups (63.6% in group A and 72.2% in group B), with hyperbilirubinemia and metabolic alterations representing the most frequently reported events. These findings are consistent with prior literature, which attributes atazanavir-associated hyperbilirubinemia to the inhibition of UDP-glucuronosyltransferase 1A1 (UGT1A1), a mechanism well-documented in protease inhibitor-based regimens [21]. Interestingly, recent data from the European Society of Cardiology (ESC) guidelines suggest that mild-to-moderate unconjugated hyperbilirubinemia, akin to that observed in Gilbert syndrome, may exert a protective effect against atherosclerotic cardiovascular disease, particularly in White individuals and women [22]. Nevertheless, this potential benefit does not mitigate the broader metabolic toxicity associated with protease inhibitors. These effects are thought to be mediated by oxidative stress and disruption of the ubiquitin-proteasome system [23]. Moreover, protease inhibitor use among PLWH has been linked to a higher burden of cardiometabolic complications, including diabetes, coronary artery disease, reduced left ventricular ejection fraction, and elevated pulmonary artery systolic pressure, and is associated with increased cardiovascular mortality and higher rates of 30-day readmission for heart failure [24].

As expected in a long-term treated cohort, a high prevalence of drug resistance mutations was observed, encompassing exposure to multiple antiretroviral classes, including older NRTI combinations used in the late 1990s [25,26]. Notably, INI resistance mutations were rare, with only one individual in each group harboring a clinically relevant mutation (5.0% vs. 7.7%, p = 0.751). This finding aligns with national and international data demonstrating a consistently low prevalence of INI resistance, even among treatment-experienced populations [27,28]. These results reinforce the pivotal role of INIs as the backbone of modern ART, as endorsed by international guidelines [3,29].

The central finding of this study concerns the crucial role of patient preference in routine clinical decision-making. Despite a comparable prevalence of ongoing ADRs between groups (63.6% in group A and 72.2% in group B, p = 0.564) and eligibility for simplification to an INI-based STR, a substantial proportion remained on OUT-ART. Specifically, 17.5% of individuals declined a switch due to self-reported intolerance to INIs, despite the absence of confirmed allergic reactions, while 37.5% expressed a general unwillingness to modify their current regimen. This highlights a challenge in HIV care: the significant impact of the patient on therapeutic decision-making, particularly in aging or treatment-experienced populations prone to therapeutic inertia. The integration of patient-reported outcomes (PROs) has been shown to enhance person-centered care by aligning clinical strategies with individual preferences [30]. Future efforts should prioritize tailored PRO use, on-site completion, and shared decision-making in accordance with current international guidelines [3].

Our study has several limitations that should be acknowledged. First, the small sample size limits statistical power and reduces the ability to detect subtle but potentially clinically relevant associations. Moreover, as this is a pilot study, the sample size was not determined through a formal power calculation, which further restricts the generalizability of the findings and underscores the exploratory nature of the analyses. Second, the observational and cross-sectional nature of the study precludes any causal inference and restricts the ability to assess the temporal relationship between variables. Furthermore, the absence of longitudinal follow-up data hinders the evaluation of long-term outcomes, particularly in the context of potential ART regimen switches. Despite these limitations, the study possesses important strengths. It offers a comprehensive and detailed clinical, virological, and immunological characterization of a real-world cohort of PLWH who have experienced prolonged exposure to OUT-ART regimens. Additionally, the study addresses an often-overlooked yet persistently present subgroup within HIV care, providing valuable insight into the challenges of treatment optimization in complex, treatment-experienced patients.

## Conclusions

This study offers a real-world description of a long-treatment-experienced cohort of PLWH who continue on OUT-ART regimens despite being clinically eligible for therapeutic simplification. Although high rates of drug resistance mutations and persistent mild-to-moderate biochemical ADRs have been observed, these factors have not prompted a transition to an INI-based STR, despite its feasibility. Our findings suggest that a combination of clinical stability, treatment familiarity, and patient-reported preferences may contribute to the maintenance of current regimens. These observations underline the importance of structured discussions between clinicians and patients to explore therapeutic options and ensure alignment between clinical indications, patient priorities, and evolving guideline recommendations.

## Appendices

Appendix A 

**Table 4:** Predictors of STR Ini switch feasibility, univariate logistic regressions Variables explication: “Reason to maintain OUT-ART” 0 = Intolerance to INI 1 = patient will; “Gender” 0 = Male, 1 = Female; “Months on OUT-ART” = number of months in outdated regimen; “Daily Pills” = number of daily pills; “Age” 0 = under 65 yo, 1 = over 65 yo; “Viral Load Zenith” and “CD4 Nadir” are continuous variables normalized between 0 (min) and 1 (max) Other variables are binomial (yes vs no)

Variable	OR	95% CI Low	95% CI Up	p-value
Ongoing ARDs	0.5292	0.1468	1.9075	0.3326
Previous Viral Failures	0.7274	0.1838	2.8787	0.6503
Previous Blips	0.5782	0.1673	21.9985	0.3868
Multimorbidity	0.5604	0.1621	1.9379	0.3625
Months on OUT-ART	2.67633	0.3182	22.4583	0.3652
Gender	0.7822	0.2159	2.8347	0.7099
Viral Load Zenith	2.0186	0.2589	15.7376	0.503
CD4 Nadir	0.8696	0.0543	13.9284	0.9215
Daily Pills	1.5576	0.6143	3.9495	0.3509
Potus	0	0	Inf	0.9999
Smoking	1.0587	0.2318	4.8343	0.9422
Age	1.1383	0.198	6.5447	0.8853
Reason to maintain OUT-ART	71.193	3.5063	1445.511	0.0075

Appendix B 

**Table 5:** Predictors of STR Ini switch feasibility, multivariate logistic regression Odds ratios (OR) are adjusted for covariates included in the reduced logistic model. Variables explication: “Reason to maintain OUT-ART” 0 = Intolerance to INI 1 = patient will; “Gender” 0 = Male, 1 = Female; “Months on OUT-ART” = number of months in outdated regimen; “Daily Pills” = number of daily pills; “Age” 0 = under 65 yo, 1 = over 65 yo; “Viral Load Zenith” and “CD4 Nadir” are continuous variables normalized between 0 (min) and 1 (max) Other variables are binomial (yes vs no)

Variable	OR	95% CI Low	95% CI Up	p-value
Ongoing ADRs	0.5372	0.0069	41.7676	0.7868
Previous Viral Failures	0.4005	0.0163	9.8505	0.5769
Previous Blips	0.1649	0.0113	2.4032	0.1916
Multimorbidity	0.8795	0.0087	88.9895	0.9576
Months on OUT-ART	1.3246	0.0142	123.9019	0.9042
Gender	3.6177	0.1587	82.4896	0.4264
Viral Load Zenith	29.2602	0.0061	139500.1	0.4493
CD4 Nadir	0.4669	0.0005	451.9289	0.8318
Daily Pills	12.1963	0.1026	1449.804	0.3279
Potus	0.6095	0.0008	469.1244	0.8847
Smoking	0.5999	0.0166	21.6247	0.7819
Age	0.1246	0.002	7.6229	0.3333
Reason to maintain OUT-ART	581.8069	2.5254	134035.4	0.0279
